# Clinical significance of detecting CSF-derived tumor cells in breast cancer patients with leptomeningeal metastasis

**DOI:** 10.18632/oncotarget.23597

**Published:** 2017-12-21

**Authors:** Xuelu Li, Yuan Zhang, Jinlei Ding, Min Wang, Na Li, Hui Yang, Kainan Wang, Dandan Wang, Peter Ping Lin, Man Li, Zuowei Zhao, Pixu Liu

**Affiliations:** ^1^ The Second Affiliated Hospital, Institute of Cancer Stem Cell, Dalian Medical University, Dalian, China; ^2^ Department of Breast Surgery, The Second Affiliated Hospital, Dalian Medical University, Dalian, China; ^3^ Institute of Cancer Stem Cell, Cancer Center, Dalian Medical University, Dalian, China; ^4^ Cancer Institute, The Second Affiliated Hospital, Dalian Medical University, Dalian, China; ^5^ Department of Oncology, The Second Affiliated Hospital, Dalian Medical University, Dalian, China; ^6^ Cytelligen, San Diego, California, USA; ^7^ College of Pharmacy, Dalian Medical University, Dalian, China

**Keywords:** breast cancer, leptomeningeal metastasis, cerebrospinal fluid-derived tumor cell, SE-i•FISH

## Abstract

Despite marked advances in breast cancer therapy, breast cancer-associated leptomeningeal metastasis (LM), a particularly aggressive syndrome with multifocal seeding of the leptomeninges by tumor cells, still carries an abysmal prognosis. A major problem with breast cancer LM surveillance is the lack of an effective and sensitive means to track dynamic changes of the disease. Cytology detection of cerebrospinal fluid (CSF) is considered the gold standard for LM diagnosis but has a high false-negative rate with a limited sensitivity. Here we applied subtraction enrichment and immunostaining-fluorescence *in situ* (SE-i•FISH) method, a technique previously used for isolating circulating tumor cells (CTCs) from the peripheral blood, to detect, enumerate, and track cerebrospinal fluid-derived tumor cells (CSFTCs) in CSF samples from 8 breast cancer patients. Comparing with cytology test, we found SE-i•FISH method can accurately and feasibly detect CSFTCs for the diagnosis of breast cancer-associated LM and monitor the disease progression. We also isolated and cultured CSFTCs from these cancer patients and performed genomic sequencing on CSFTCs of two patients. Genomic analysis of CSFTCs against corresponding archival primary breast tumors revealed clonal relationships with some ongoing evolution. Further drug sensitivity test on cultured CSFTCs based on genomic analysis data helped identify promising treatment options for the patient tested. Together, our results suggest that CSFTCs detection using SE-i•FISH platform could serve as a sensitive and accurate method to make the diagnosis and a promising approach to monitor tumor dynamics and treatment response for breast cancer-associated LM.

## INTRODUCTION

The cerebrospinal fluid (CSF) lies in between leptomeninges, which are the membranes lining the brain and spinal cord [1]. Leptomeningeal metastasis (LM), also called neoplastic meningitis and carcinomatous meningitis, is a particularly aggressive syndrome with very high mortality [2, 3, 4]. LM from breast cancer has been reported to occur in approximately 2%–5% of cases over the natural course of the disease [1, 2, 3]. With development of more sensitive diagnostic technology and better imaging modalities that allow for more accurate and earlier diagnosis, along with the improvement in effective targeted drugs that extend patients’ lives, the incidence of LM is on the rise [1, 2, 3]. Management strategies available for breast cancer patients with LM are limited without published guidelines, only including whole brain radiation therapy (WBRT), surgery, stereotactic radiosurgery (SRS), intrathecal and systemic chemotherapy [2]. Treatment remains difficult and little is known about the particularly aggressive metastatic process. Therefore, it is urgent to develop a feasible and reliable method to help diagnose LM and monitor the treatment response of this type of breast cancer patients.

The best test for LM detection is to obtain CSF samples by performing a lumbar puncture and subsequently the CSF is examined for cancer cells using papanicolaou staining. Unfortunately, the sensitivity of cytology examination of LM is at approximately 50% and 85–90% for the first and the second puncture, respectively [2]. As the sensitivity and methods for detection of LM are relatively limited, we set out to establish a subtraction enrichment and immunostaining-fluorescence *in situ* hybridization (SE-i•FISH) platform for isolation and functional analysis of cerebrospinal fluid–derived tumor cells (CSFTCs) with the purpose to improve the diagnosis, reduce false negative results, and gain molecular understanding of this disease. The SE-i•FISH platform has been previously used for enrichment and identification of circulating tumor cells (CTCs) from blood, which is irrespective of the specific surface marker expression and cell size variation. The unbiased enrichment and identification of highly heterogeneous CTCs/CSFTCs lead to a higher tumor cell detection efficiency compared to the conventional CSF cytological examination.

In this study, we employed SE-i•FISH platform to detect and isolate CSFTCs from 8 breast cancer patients with LM, an approach by which we can enrich non-antibody perturbed native tumor cells suitable for primary cell culture and additional biological analyses. We found that enumeration of CSFTCs could help make a quick and accurate diagnosis and evaluate treatment response of LM. Furthermore, genomic and functional analyses of cultured CSFTCs may help identify mutated drug targets and assist preclinical personalized drug sensitivity test.

## RESULTS

### Patient characteristics

Eight breast cancer patients have been diagnosed with leptomeningeal metastases (LM) (Table 1). The cerebrospinal fluid (CSF) samples were obtained from these patients at the initial diagnosis of LM and before intrathecal chemotherapy. Cerebrospinal fluid–derived tumor cells (CSFTCs) were observed in all patients by standard cytologic detection of malignant cells in CSF. Neurological symptoms, such as headache, vision changes and nausea, were present in eight patients with LM. Seven of eight cases (87.5%) showed positive magnetic resonance imaging at the time of initial LM diagnosis. All patients had presented multi-organ metastases before diagnosis of LM, mainly including bone and visceral metastases. The median age at diagnosis of primary breast cancer was 37 years (range 27–51). The median interval between the diagnosis of primary breast tumor and LM was 4.5 years (range 0.5–9 years), and the median overall survival time after the diagnosis of LM was 10.5 months (range 0.8–13 months). Eight patients exhibited progressive disease and died thereafter. The causes of death were all cancer-related.

**Table 1:** Summary of breast cancer patients with leptomeningel metastases

No.	Age *(PMT)*	Molecular classification of PMT				Age *(LM)*	Survival Time *(LM to Death)*	CSFTC count per 1 ml	Time from single cell to cluster
		*ER*	*PR*	*HER2*	Ki67				
NM01	27	-	-	-	30%	28	9 months	195	21 days
NM02	34	-	-	-	50%	43	1 year	-	No cluster
NM03	40	50%	-	-	30%	47	20 days	67	No cluster
NM04	46	-	-	-	80%	48	6 months	328	12 days
NM05	33	-	-	-	50%	39	8 months	-	No cluster
NM06	43	-	-	-	40%	49	11 months	122	No cluster
NM07	51	<1%	<1%	-	40%	51	1 year	218	10 days
NM08	32	70%	50%	-	20%	35	13 months	-	No cluster

Note: PMT, primary tumor. LM, leptomeningel metastasis. ‘-’, the data is not available.

**Figure 1:**
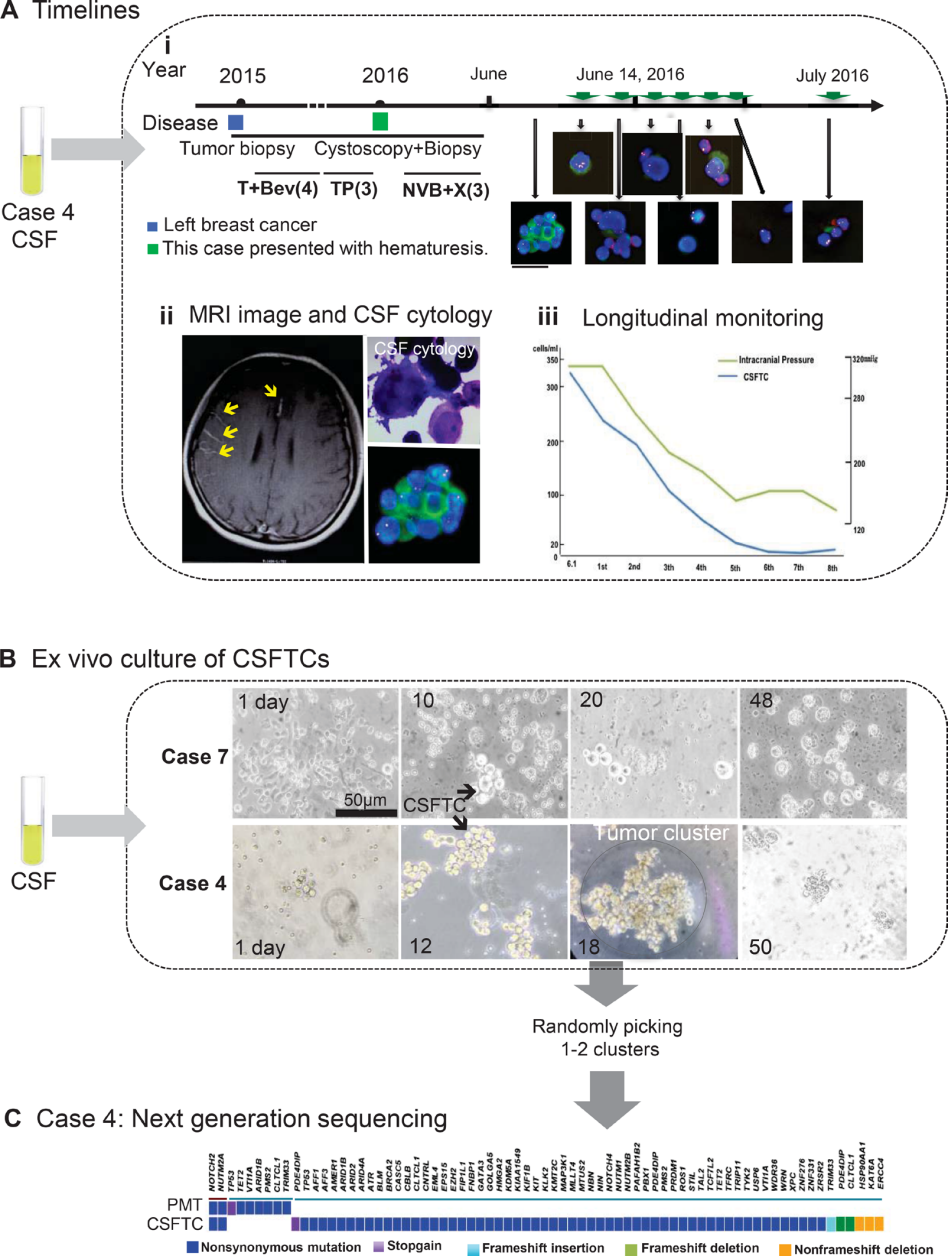
Overview of SE-i•FISH platform for dynamic monitoring and *ex vivo* culture of CSFTCs (**A**) The treatment timelines received by the patient (NM04). (i) From 2015 until 2016, the patient received four cycles of adjuvant T+Bev (Paclitaxel/Bevacizumab) and three cycles of TP (Paclitaxel/DDP) chemotherapy. In January 2016, the patient presented with hematuresis. Following urethrocystoscopy biopsy, the patient received three cycles of NVB+X (Vinorelbine/Capecitabine). The treatment had to stop as she presented with excruciating headache and vomiting. On the basis of the diagnosis of leptomeningeal metastases by MRI and CSF cytology, the patient received regularly intrathecal methotrexate and cytarabine. Green arrows mean intrathecal chemotherapies. The immunofluorescent staining for cytokeratin (CK, green), chromosome 8 (Yellow), CD45 (Red), and nuclei (DAPI, blue). (ii) Representative images of MRI and CSF cytology. (Upper right panel) Light microscopic imaging with Papanicolaou staining. (iii) The number of CSFTCs and the intracranial pressure are shown in the right panel. (**B**) *Ex vivo* culture of CSFTCs. 5–10 ml CSF were obtained from patients and then enriched. CSFTCs were cultured in a medium without serum under suspension culture conditions. The CSFTCs of NM04 and NM07 could be expanded in short term as indicated. Scale bar, 50 μm. (**C**) At Day 18, the tumor clusters of case NM04 were randomly picked. The exome sequencing was performed on DNA extracted from CSFTCs and the paired primary tumor. The distribution of somatic mutations detected by exome sequencing is presented in a heat map.

Besides the ‘gold standard’ cytologic detection, we also employed a recently reported SE-i•FISH platform to monitor the number of CSFTCs during intrathecal chemotherapy (Figure 1A) [5, 6]. The concentrations of CSFTCs in threes patients (NM01, NM04, NM07) whose CSFTCs tended to generate tumor clusters in culture were higher than those from patients whose CSFTCs did not generate tumor clusters (Table 1 and Figure 1). We observed that the doubling time of CSFTCs *in vitro* culture ranged from 10–21 days (Table 1).

### Dynamic monitoring of CSFTCs

**Figure 2:**
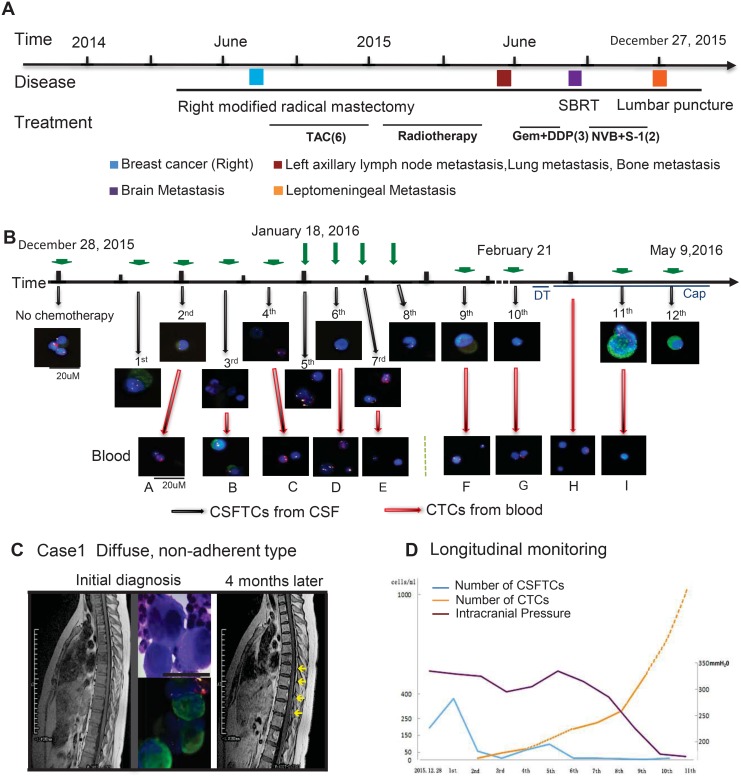
Treatment timelines and longitudinal monitoring of CSFTCs (**A**, **B**) The treatment timelines received by the patient (NM01). From 2014 until 2015, the patient received six cycles of postoperative adjuvant TAC (Taxotere/Doxorubicin/Cyclophosphamide) chemotherapy, radiotherapy and three cycles of Gem+DDP (Gemcitabine/Cisplatin). In August 2015, brain metastases were detected and the patient underwent SBRT (Stereotactic body radiotherapy). Three cycles of NVB+S-1 (Vinorelbine/Tegafur) were given to the patient and the treatment had to stop as she presented with excruciating headache and vomiting. Leptomeningeal metastases were diagnosed by CSF cytology using Papanicolaou staining (**C**). In 2016, she received regularly intrathecal chemotherapy (Methotrexate/Cytarabine). Representative images of CSFTCs and CTCs are also shown. Green arrows mean intrathecal chemotherapies. Red arrows mean that we simultaneously monitored CTCs in blood. The immunofluorescent staining for cytokeratin (CK, green), chromosome 8 (Yellow), CD45 (Red), and nuclei (DAPI, blue). (C) Initial MRI examination did not show leptomeningeal metastases (left), whereas it appeared after 4 months. The yellow arrowheads are pointing to the leptomeningeal metastases (right). Dynamic changes of intracranial pressures (purple), CSFTC (blue) and CTC (yellow) counts as indicated in (**D**).

To evaluate the feasibility of applying the SE-i•FISH platform to monitor treatment response, we retrospectively examined if the number of CSFTCs correlated positively with the intracranial pressure and neurological symptoms of the same patients. We found that there was a remarkable concordance between the number of CSFTCs and the aforementioned clinical markers (Figures 1A, 2D; Supplementary Tables 1, 2). However, there was no such correlation between sensitivity to treatment response and the routine CSF cytology that only detected whether cancer cells were existent. These findings indicated that CSFTCs detection by the SE-i•FISH platform might offer breast cancer LM patients a good opportunity to monitor the disease progression.

### CSFTCs enriched by SE-i•FISH platform can be cultured *in vitro*

We next enriched tumor cells (subtraction enrichment, as reported in *Materials & Methods*) from the chemotherapy-naive CSF to investigate whether CSFTCs could proliferate *in vitro*. We cultured CSFTCs in a medium without serum under suspension culture conditions so as to inhibit cell differentiation and maintain tumor cells at an undifferentiated state [7, 8, 9]. The CSFTCs from case NM01, NM04 and NM07 could be expanded and grew *in vitro* for at least seven weeks, but they gradually underwent senescence after that period (Figure 1B). However, attempts of CSFTCs *ex vivo* culture from the other patients were unsuccessful.

To identify potential actionable mutations and the origin of CSFTCs, we performed next generation sequencing on DNA extracted from CSFTCs and the matched primary breast tumors. For case NM01 and NM04, the genomic DNA exhibited sufficiently high purity, but the DNA libraries of primary tumor from case NM07 were not of sufficient quality. Therefore, the exome sequencing was only performed on NM01 and NM04. In case NM04, we identified non-synonymous SNVs of NOTCH2 and NUTM2A genes shared in both CSFTCs and the matched primary tumor and some private gene mutations either in CSFTCs or in the matched primary tumor, suggesting the common origin of the primary tumor and CSFTCs and ongoing clonal evolution during disease progression (Figures 1C, 4A; Supplementary Tables 3, 4). We also obtained similar findings in case NM01 that we will present later in details. Taken together, our results demonstrated that the SE-i•FISH platform could be used to isolate and culture CSFTCs and assist the molecular profiling of breast cancer associated LM.

**Figure 3:**
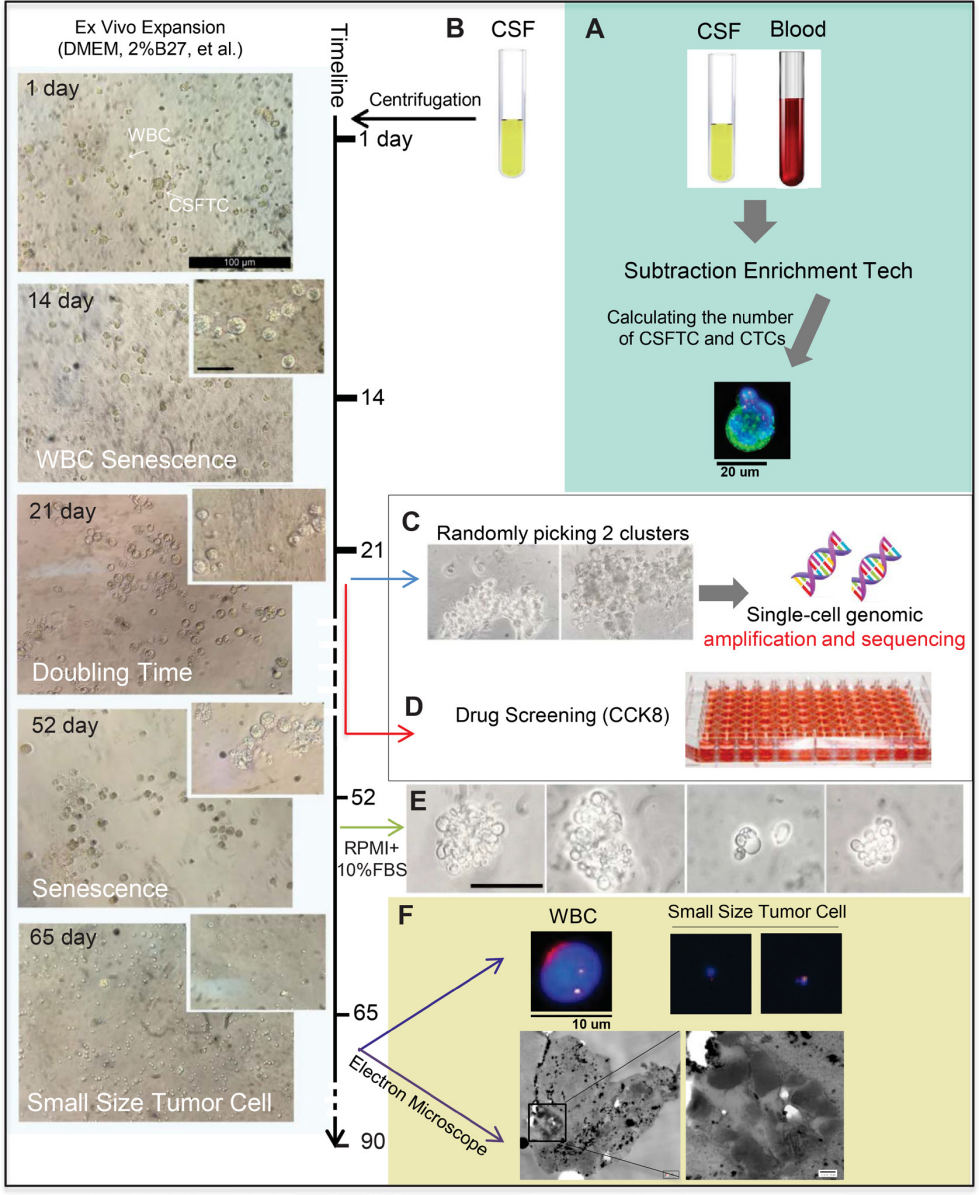
*Ex vivo* expansion of CSFTCs from case NM01 The left panel showed representative images of *ex vivo* culture of CSFTCs. The CSFTCs could be expanded for more than seven weeks, but after 90 days they gradually underwent senescence. (**A**) The number of CTCs in blood and CSFTCs were recorded by the SE-i•FISH platform. (**B**) Cerebrospinal fluid was collected and centrifuged. Subsequently, CSFTCs were enriched by subtraction enrichment (SE) technology. (**C**) At Day 21, cultures appeared tumor clusters, and then two tumor clusters were picked for single-cell genomic amplification and exome sequencing. (**D**) *Ex vivo* drug sensitivity test was assessed by CCK8 assay. (**E**) The morphological changes of CSFTCs were observed, when the special non-serum medium was switched to the medium with 10% FBS. At Day 65, we observed the small size cells. Representative images of immunofluorescence (up) and scanning electron microscope (down) are shown in (**F**).

**Figure 4:**
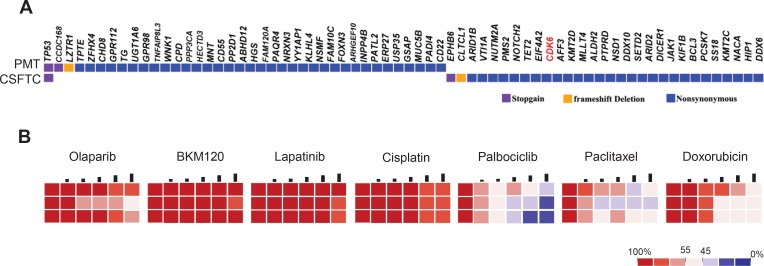
Drug sensitivity on CSFTCs as a potential predictive tool for treatment response (**A**) The distribution of somatic mutations detected by exome sequencing was presented in a heat map. (**B**) Heatmaps represent cell viability after treatment of selected anticancer drugs. Each concentration represents a 2-fold increase from the previous dose, with each concentration tested in triplicate.

### SE-i•FISH platform used in a typical patient

Here we described the case of a 27-year-old female (NM01) who underwent a right modified radical mastectomy and then received a series of adjuvant therapies (Figure 2A). In December 2015, the postoperative chemotherapy had to be stopped for this patient due to excruciating headache and vomits, indicative of intracranial hypertension and likely leptomeningeal metastasis (Figure 2B). Initial MRI examination did not show leptomeningeal metastases that were diagnosed by CSF cytology (Figure 2C). The levels of tumor biomarkers (CA125, CA153 and CA199) in blood were within the normal range throughout the whole course of treatment, which was in discordance with the patient's condition. CSF cytology test was positive but it only detected whether cancer cells were existent without providing information about the dynamic change of tumor cells in the CSF. To this end, we monitored the number of CSFTCs and circulating tumor cells (CTCs) in blood during intrathecal chemotherapies using SE-i•FISH platform (Figures 2B, 2D and 3A). Retrospectively, we found there was a correlation between the number of CSFTCs and clinical symptoms: when the patient presented with headache alleviation at the third intrathecal chemotherapy, we detected the number of CSFTCs as low as 12 per ml of cerebrospinal fluid (Figure 2D, Supplementary Table 1); when the headache of this patient became worse at the fifth intrathecal chemotherapy, we found the number of CSFTCs increased to as many as 95 per ml. As there was no intravenous chemotherapy given in the course of intrathecal chemotherapy, the number of CTCs in blood increased gradually during this treatment period (Figure 2D). Our findings indicated that this method could dynamically monitor clinical response of the patient to intracranial chemotherapy and progression of the disease.

We next expanded CSFTCs from case NM01 in the special non-serum medium for 21 days and performed exome sequencing on CSFTCs and the matching primary breast tumor (Figure 3B, 3C). In addition, we carried out the drug sensitivity assay of these cultured CSFTCs (Figure 3D). Interestingly, we observed the morphological changes of CSFTCs after 52 days culture, some of which were small size cells (Diameter≈3–4 μm), when comparing with those cultured in the medium with 10% FBS (Figure 3E, 3F). A recent study showed that the existence of small sized tumor cells in the blood was correlated well with tumor metastatic potential [16]. We further characterized these small sized tumor cells from three aspects (Figure 3F): (1) FISH (chromosome 8) analysis indicated that these cell were human origin; (2) electron microscope observation showed the existence of organelles within the cells and the size of cells (about 3–4 μm); (3) 3D culture assay revealed that these cells proliferated in a 3D condition (Supplementary Figure 1), proving their ability of growth *in vitro*.

### Drug sensitivity test on CSFTCs as a potential predictive tool in selection of treatment regimen

We further characterized CSFTCs of case NM01 at the genome level by performing exome sequencing on CSFTCs and the paired primary tumor to look for actionable mutations that can be targeted. We identified somatic mutations in 61 genes with the primary tumor and CSFTCs carrying 30 private mutations respectively; one SNV of TP53 was shared by both the primary tumor and CSFTCs, suggesting heterogeneity and ongoing clonal evolution of the tumor cells examined in this case (Figure 4A, Supplementary Table 4).

We then tested the drug sensitivity of cultured CSFTCs of case NM01 to several most commonly clinically used chemotherapy drugs (cisplatin, paclitaxel, doxorubicin) and drugs for targeted inhibition (Figure 4B, Supplementary Table 5). It had been reported that breast cancer cells with p53 mutations exhibited impaired DNA damage response and increased sensitivity to poly-ADP ribose polymerase (PARP) inhibitors [10]; in addition, INPP4B was proposed as a tumor suppressor to modulate PI3K signaling and lapatinib was a tyrosine kinase inhibitor that could prevent triple-negative breast cancer metastasis to the brain [11, 12, 13]. Based on these previous studies and our sequencing data, we chose to use PARP inhibitor Olaparib, PI3K inhibitor BKM120, tyrosine kinase inhibitor lapatinib. Several studies reported that Palbociclib could overcome CDK6 mutations (D163G, H100L) [14, 15]. We proposed CDK4/6 inhibitor palbociclib that would target CDK6 gene mutation (V77G) carried by CSFTCs in this case. Of all the drugs tested, selective CDK4/6 inhibitor palbociclib displayed the best anticancer effect, while the others were ineffective or just showed modest cytotoxic effects on CSFTCs viability test (Supplementary Table 5), suggesting that target inhibition of CDK6 in this patient would be an effective treatment option against CSFTCs (Figure 4). Together, our data indicated that *ex vivo* culture of CSFTCs may serve as a predictive model for assessing potential clinical therapeutic paradigms.

## DISCUSSION

Here we describe an effective method for monitoring and *ex vivo* expansion of CSFTCs from breast cancer patients with leptomeningeal metastases. We found that there was a remarkable concordance between the number of CSFTCs and the aforementioned clinical markers such as intracranial pressure, the result of CSF cytology and clinical symptoms. On the other hand, the exome sequencing on CSFTCs identified potential actionable mutations that could be used to assist drug sensitivity test for each individual patient. All these data supported the notion that the SE-i•FISH platform could be applied to detect tumor cells in the CSF and more importantly, monitor the disease progression and treatment response.

Although CSF cytology is the current gold standard for LM diagnosis, this examination is notoriously insensitive [1, 2, 16]. Our results showed that the number of CSFTCs was concordant with headache alleviation and the dynamic changes of intracranial pressure. Lee *et al*. also reported the similar observation that changes in CSFTC counts predicted treatment response in seven breast cancer patients with leptomeningeal metastases [17].

The integrated SET-iFISH platform has been applied to detect and characterize CTCs in gastric, lung, and esophageal cancer patients [5, 6]. In this study, we applied the SE-i•FISH platform for CSFTC enrichment on CSF samples from 8 breast cancer patients with leptomeningeal metastasis and cultured all of the CD45 negative remaining cells in non-adherent culture conditions. Only three lines of CSFTCs, namely NM01, NM03 and NM07, could be expanded *ex vivo* with relatively high concentration of tumor cells in the cerebral spinal fluid (>195 per ml), suggesting a requirement for certain tumor cell concentration for successful *ex vivo* culture of CSFTCs. For the specific CSFTCs from case NM01, we switched from the special non-serum medium to the medium with 10% FBS after seven weeks of cell culture and observed the morphological changes of CSFTCs, which were the small size cells. In addition, we also independently isolated small size tumor cells from blood and CSF samples of this patient at different time points. Our findings were consistent with the study from Chen *et al*. who reported that small nuclear CTCs were correlated with the tumor metastases [16, 18]. However, the clinicopathological features and molecular characteristics of these small size tumor cells remains to be identified.

The next generation sequencing identified the shared non-synonymous SNVs in both CSFTCs and the matched primary tumor, suggesting the common origin of these tumor cells. In contrast, we found some private gene mutations either in CSFTCs or in the matched primary tumor. The heterogeneous patterns of these genetic mutations from primary tumor and CSFTCs suggested the ongoing clonal evolution during disease progression. In addition, drug sensitivity test and mutation analysis may help identify effective therapeutic strategies for each individual patient [7, 8, 9, 19, 20, 21]. We noted that selective CDK4/6 inhibitor palbociclib exhibited the best anticancer effect with reference of the CDK6 gene mutation (V77G) carried by CSFTCs in case NM01. Our preclinical assessment of therapeutic options provides a potential effective treatment regimen based on the *ex vivo* drug sensitivity assay in combination with mutation analysis of this patient, a practice which needs to be validated in subsequent clinical treatment.

Together, CSFTC detection using SE-i•FISH platform provides a new approach to measure CSFTC levels in a dynamic manner. Changes in the CSFTC counts correlate well with LM disease state and therefore could serve as a predictor to monitor disease progression and treatment response of LM patients. In addition, the isolation and *ex vivo* culture of CSFTCs will facilitate molecular characterization of CSFTCs and their subtypes, as well as understanding the pathological nature of LM. The present study suggests that this platform may be commonly used in treating cancer patients with LM in the future. Further comprehensive profiling of CSFTCs at genomic, transcriptomic or proteomic levels in combination with functional studies of these tumor cells will help guide diagnosis, design of treatment regimes, drug response and prognosis for breast cancer LM patients.

## MATERIALS AND METHODS

### Patients and samples

Clinical samples were obtained from patients with breast cancer who were diagnosed with leptomeningeal metastases and were treated at the Second Hospital of Dalian Medical University between January 2013 and December 2016 (Table 1). T1 weighted enhancement magnetic resonance imaging (MRI) was the standard diagnostic procedure for patients with leptomeningeal metastases. Meningeal metastasis was also confirmed by cerebrospinal fluid (CSF) cytology. CSF samples were centrifuged. Subsequently, the smear was made by direct application of slide. Before the slide dried, it was fixed and stained according to Papanicolaou staining instruction. Eight patients provided their written informed consent. The study was approved by the Second Hospital of Dalian Medical University, and was performed according to the Declaration of Helsinki.

### Enumeration of CSFTCs and CTCs in blood

Experiment was performed according to the product manufacture's instructions (Cytelligen, San Diego, CA, USA). Briefly, the peripheral blood and cerebrospinal fluid were collected into Cytelligen tubes containing ACD anti-coagulant (Becton Dickinson, Franklin Lakes, NJ, USA) and centrifuged at 450 × g for 5 min. Supernatant was collected and incubated with anti-WBC (CD45) and endothelial cell immunomagnetic beads at room temperature for 20 min with gentle shaking. Subsequently, solution was subjected to magnetic separation. Bead-free solution was spun at 500 × g for 2 min. The sedimented cells containing rare cells were mixed with 100 μl cell fixative, and applied onto the formatted and coated slides for subsequent iFISH analysis. Air dried samples were suitable for subsequent analyses, including immunofluorescent staining (CK18, DAPI and CD45) and immunostaining-fluorescence *in situ* hybridization (centromere 8) (i•FISH). Images of cells were visualized with a fluorescence microscope (Olympus, Tokyo, Japan). CSFTCs were identified as DAPI+/CD45–/CK+ or DAPI+/CD45−/CK− with aneuploidy or polyploidy of chromosome 8.

### Cell culture

Subtraction enrichment (SE) was performed according to the product manufacture's instruction (Cytelligen, San Diego, CA, USA). Enriched tumor cells were grown in 24-well low-attachment plates with medium consisting of RPMI-1640 medium (Gibco, Carlsbad, CA) supplemented with EGF (20 ng/ml), basic FGF (20 ng/ml), B27 (2%), Insulin (5 mg/ml), Hydrocortisone (0.5 mg/ml) and Antibiotic-antimycotic (1%) (Life Technologies) [7, 8, 9]. Culture medium was changed every 4 days under microscopic monitoring of CSFTCs. 3D cell culture experiments were performed as described previously [22]. Briefly, cancer cells were seeded on 96-well plates coated with 100 μl Matrigel (BD Biosciences). Cells were grown in the above-described RPMI-1640 medium supplemented with 2% Matrigel. Cells were incubated at 37°C in a 5% CO_2_-containing atmosphere.

### Next generation sequencing analysis

The FFPE primary tumor tissues and normal oral epithelial cells were disaggregated, and gDNAs were isolated using QiaAmp FFPE DNA Mini kit and QiaAmp DNA Mini kit (Qiagen). The extracted genomic DNA was sequenced on the Illumina Hiseq2000 platform. To remove artifacts likely caused by cytosine deamination due to fixation/long-term storage of FFPE samples [23] and the low tumor cell content of samples, the following filtering criterion was applied: somatic mutations were called when supported by at least five reads, representing at least 10% of the total reads.

CSFTCs were disaggregated. Subsequently, single-cell genomic amplification was performed using REPLI-g Single Cell Kit (Qiagen). The target capture was performed using a custom Agilent SureSelect assay for 576 cancer-related genes targeting an average coverage of 500× for the panel (Gentalker, China). To remove the presence of a low tumor cell content of samples, the following filtering criterion was applied: somatic mutations were called when supported by at least five reads, representing at least 5% of the total reads.

### Drug sensitivity assay

Cells were seeded one day prior to treatment in 96-well plates at 2000 cells per well. Each drug was added at 5 different concentrations. Cell viability was tested after 4 days of drug exposure using CCK-8 assay according to the product manufacturer's instructions (Dojindo Molecular Technologies), and was normalized to corresponding vehicle controls.

### Scanning electron microscopy

Small size tumor cells were collected and centrifuged at 1050 × g for 5 min. These cells were embedded into 2.5% agar and fixed in glutaraldehyde. The cells were observed with TEM at 120 KV using ATM Camera System.

## SUPPLEMENTARY MATERIALS FIGURES AND TABLES






