# Supplementary material for: Clinical significance of detecting CSF-derived tumor cells in breast cancer patients with leptomeningeal metastasis
Source: Oncotarget. 2017 Dec 21;9(2):2705–14. doi: 10.18632/oncotarget.23597 (PMC5788671; doi:10.18632/oncotarget.23597)
Supplement: Supplementary file 1 [file oncotarget-09-2705-s001.pdf]

Clinical significance of detecting CSF-derived tumor cells in breast cancer patients with leptomeningeal metastasis

SUPPLEMENTARY MATERIALS

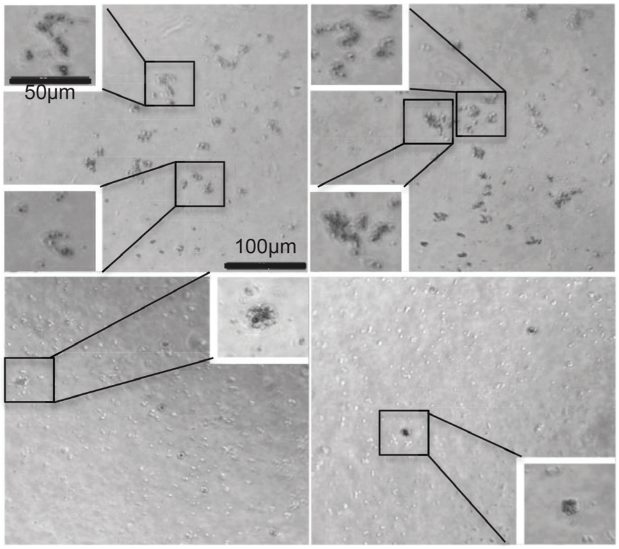

Supplementary Figure 1: Representative images of small size tumor cells cultured in 3D Matrigel with different mediums.

Supplementary Table 1: The dynamic changes of the monitoring indicators in case NM01

Date		12.28	1st	2nd	3rd	4th	5th	6th	7th	8th	9th	10th	4.6	4.21	5.9
CSF	*CSFTC*	195	373	54	12	60	95	12	10	6	3	11	/	38	30
	ICP	330	325	320	290	300	330	310	280	220	170	165	/	165	160
	Cyto	Cluster	Lots	Few	N	Few	Few	Few	/	N	N	N	/	Few	N
CTC(Blood)		/	/	12	44	68	/	189	226	/	298	510	1031	/	/

Supplementary Table 2: The dynamic changes of the monitoring indicators in case NM04

Date		6.1	1st	2nd	3rd	4th	5th	6th	7th	8th	unit
CSF	*CSFTC*	328	243	201	119	68	28	12	10	16	Cells/ml
	ICP	310	310	260	220	200	170	180	180	160	mmHg
	Cyto	Cluster	Lots	Few	Few	Few	N	Few	Few	N	-

Supplementary Table 3: The somatic mutations identified in case NM04. See Supplementary_Table_3

Supplementary Table 4: The somatic mutations identified in case NM01. See Supplementary_Table_4

Supplementary Table 5: Drug sensitivity of cultured CSFTCs

	DMSO	0.3125	0.625	1.25	2.5	5	(μM/ml)
	0.99	0.91	0.87	0.88	0.77	0.78	
Olaparib	0.995	0.88	0.63	0.69	0.68	0.54	
	1.00	0.90	0.89	0.88	0.79	0.69	
	0.99	0.96	0.93	0.93	0.91	0.85	
BKM120	0.99	1.00	0.96	0.92	0.89	0.83	
	0.99	0.93	0.92	0.92	0.89	0.87	
	0.99	0.67	0.46	0.66	0.46	0.31	
Pabociclib	0.99	0.66	0.55	0.45	0.33	0.20	
	0.99	0.66	0.51	0.31	0.25	0.22	
	0.98	0.95	0.88	0.87	0.87	0.86	
Lapatinib	1.00	0.94	0.92	0.90	0.85	0.84	
	1.00	0.96	0.96	0.89	0.85	0.82	
	1.00	0.830	0.638	0.592	0.430	0.466	
Paclitaxel	0.99	0.649	0.405	0.438	0.324	0.324	
	0.99	0.631	0.461	0.567	0.525	0.507	
	0.99	0.955	0.730	0.730	0.618	0.511	
Doxorubicin	0.99	0.865	0.725	0.490	0.535	0.476	
	0.99	0.935	0.822	0.490	0.490	0.478	
	0.996	0.93	0.89	0.87	0.82	0.77	
Cisplatin	0.99	0.99	0.90	0.88	0.82	0.80	
	1.00	0.95	0.93	0.90	0.82	0.77	
